# An optimum study on the laser scanning confocal microscopy techniques for BiFC assay using plant protoplast

**DOI:** 10.1186/s40529-024-00409-z

**Published:** 2024-01-09

**Authors:** Jinhong Yuan, Daiyu Li, Yi Liang, Yao Meng, Li Li, Lin Yang, Mingyue Pei, Liuchun Feng, Junhua Li

**Affiliations:** Engineering Research Center of Crop Genetic Improvement and Germplasm Innovation in Henan Province, College of Life Sciences, Henan Normal University, Xinxiang, 453007 China

**Keywords:** BiFC, Confocal microscopy, Protoplast, Preparation and observation

## Abstract

**Background:**

The bimolecular fluorescence complementation (BiFC) assay is commonly used for investigating protein–protein interactions. While several BiFC detection systems have been developed, there is a limited amount of research focused on using laser scanning confocal microscope (LSCM) techniques to observe protoplasts. Protoplasts are more susceptible to damage and instability compared to their original cell state due to the preparation treatments they undergo, which makes it challenging for researchers to manipulate them during observation under LSCMs. Therefore, it is crucial to utilize microscope techniques properly and efficiently in BiFC assays.

**Results:**

When the target fluorescence is weak, the autofluorescence of chloroplast particles in protoplasts can interfere with the detection of BiFC signals localized in the nuclear region. Spectrum analysis revealed that chloroplast autofluorescence can be excited by lasers of various types, with the highest fluorescence signal observed at around 660 nm. Furthermore, our investigation into the impact of different pipette tips on the integrity of protoplast samples indicated that the utilization of cut tips with larger openings can mitigate cell breakage. We presented a workflow of LSCM techniques for investigating protoplast BiFC and discussed the microscopic manipulation involved in sample preparation and image capturing.

**Conclusion:**

When the BiFC signals are weak, they may be affected by chloroplast autofluorescence. However, when used properly, the autofluorescence of chloroplasts can serve as an excellent internal marker for effectively distinguishing other signals. In combination with other findings, this study can provide valuable reference for researchers conducting BiFC assays and related studies.

## Background

Bimolecular fluorescent complementation (BiFC) is a noninvasive technique that utilizes the complementation of split fluorescent protein fragments to test the interaction of genes/proteins in live cells. The fluorescent protein is split at the non-conserved region, resulting in two non-fluorescent N- and C-terminal polypeptide segments. The genes of the two polypeptide segments are separately fused with a pair of target genes of interest. If the target genes interact with each other, the two polypeptide segments of the fluorescent protein may come close in space and regain fluorescence. Since the report of the BiFC technique in 2002, numerous new fluorescent proteins and variants have been developed and applied in various plant species (Heim and Tsien 1996; Hu et al. 2002; Nagai et al. 2002; Patterson and Lippincott-Schwartz 2002; Shaner et al. 2004). The fluorescent proteins can emit different light colors, including blue, cyan, green, yellow, red and others, which almost cover the entire spectral range of visible light. These offer flexible options for examining the proteins that interact within live cells (Guo et al. 2023; Hu and Kerppola 2003; Jach et al. 2006; Shyu et al. 2006).

Fluorescent proteins emit light with longer wavelengths and lower energy compared to the excitation light. This property allows for the excitation and observation of fluorescent signals in live cells using either a laser scanning confocal microscope (LSCM) or an epifluorescence microscope to localize interacting proteins. Developed in the mid-1980s, the LSCM differs from an epifluorescence microscope in that it utilizes lasers with single wavelengths as the light source rather than utilizing continuous wavelengths from a lamp. Both the excitation and emission sides of the LSCM can be adjusted to optimize spectrum settings. Furthermore, the LSCM provides higher image clarity by detecting fluorescence signals only from the focused specimen plane, thus eliminating background noise from unfocused layers.

In protoplast BiFC studies, plant cells first undergo enzyme digestion to remove their cell walls. Then, protoplasts are incubated with plasmids that have been fused with the genes of interest. Once the proteins have co-expressed in the protoplasts, their interaction and subcellular localization are investigated by observing the fluorescence within the live cells. The growth of *Arabidopsis* plants typically takes approximately three to four weeks. It takes a few days to construct fused plasmids, 6–8 h for protoplast preparation and DNA co-transfection, and 2–48 h for incubating the protoplasts to enable the expression of the BiFC proteins (Ohad et al. 2007; Yoo et al. 2007). Following transfection, samples should be promptly examined under microscopes. At present, there are numerous studies focused on the development of fluorescence proteins and BiFC vector construction. However, there is still a lack of research regarding microscopic techniques, possibly stemming from the assumption that microscopic techniques for protoplast BiFC have already been well mastered. However, the observation using LSCM relies largely on empirical evidence, which could potentially limit the applicability of the BiFC assay. Protoplasts are delicate, and their observation duration may be shortened for various reasons. For example, *Arabidopsis* protoplasts are susceptible to movement or loss of integrity after laser irradiation. For individuals unfamiliar with LSCM microscopic manipulation and parameter settings, capturing an image of satisfactory quality can be challenging. Consequently, some researchers prefer to use transfected onion bulb epidermis or *Agrobacterium*-mediated transformed leaves as materials, or opt for epifluorescent microscopes for inspection. However, these approaches may compromise certain details of intracellular signals within cells due to increased background interference caused by unfocused layers.

In this study, *Arabidopsis* protoplasts are used as the experimental material and the BiFC techniques using a LSCM are investigated. The primary goal was to analyze the variations in fluorescence signal intensities and their corresponding images. Additionally, the autofluorescence spectrum of *Arabidopsis* chloroplasts was examined, and the integrity of protoplasts when different pipette tips were used was compared. Furthermore, this study explored various factors, such as sample mounting, target protoplast screening, and emission spectrum settings that might influence the LSCM imaging of protoplasts. The findings of this study can serve as a valuable reference and provide insights for future research in this field.

## Materials and methods

### Materials

In this study, *Arabidopsis thaliana* wild type (Columbia-0) plants were utilized. The seedlings were grown at 22 °C with a photoperiod of 16 h light/8 h dark (Li et al. 2016).

The enzyme solution for transient gene transformation was prepared as previously described (Yoo et al. 2007), except that it contained 1% cellulose R10, 0.1% macerozyme (Yakult, Japan). Additionally, a DAPI solution containing 5 μg/mL 4ʹ,6-Diamidino-2-phenylindole dihydrochloride (Sigma, USA) in ddH_2_O was also prepared as previously described (Li et al. 2022).

### Preparation of the protoplast and fused plasmid

Protoplast and fused plasmid preparation, as well as transient gene expression with *Arabidopsis* mesophyll protoplasts, were conducted following the method described by Yoo et al. (2007). Briefly, slices of moderate-sized *Arabidopsis* leaves were obtained from 4-week-old seedlings. Protoplasts were collected by incubating the slices with an enzyme solution for 4 h to digest the cell walls. Simultaneously, two fusion plasmids were extracted using the Plasmid Miniprep Kit (Zoman, China), one containing the gene of interest fused with cCFP and the other with nVenus. The protoplasts obtained after enzyme digestion were washed and examined under bright-field microscopy to ensure that most of their shapes remained intact. Afterwards, they were incubated with the two plasmids in the dark at 25 °C for approximately 16 h. The interaction between the genes of interest and their intracellular localization was investigated by detecting yellow fluorescence 24 h after transfection. To localize the cell nucleus, the sample was stained with a DAPI solution for 10–20 min before inspecting the BiFC signals using a LSCM.

### The acquisition of fluorescent images

In our study, we utilized the LSCM (Leica, TCS SP8, German) equipped with an inverted microscope (Leica, DM6000, German). Fluorescence detection was conducted using two ways. The first way involved observing epifluorescence through ocular lenses. A halogen lamp was used as the light source, and the microscopic filter sets for excitation and emission were as follows: I. Exciter at 360/40 nm, dichroic mirror at 400 nm, and emitter at 425-nm (above 425 nm) for blue fluorescence; II. Exciter at 470/40 nm, dichroic mirror at 510 nm, and emitter at 515-nm for green to yellow fluorescence; III. Exciter at 538/45 nm, dichroic mirror at 580 nm, and emitter at 590-nm for red fluorescence. The second way involved exciting fluorescence using lasers with single spectral lines at wavelengths of 405 nm, 488 nm, 514 nm, 552 nm, and 638 nm as the light source. The LAS AF software (Leica) was used to display and capture the images. Prior to detecting different types of fluorescence, the excitation and emission wavelengths were adjusted accordingly. For DAPI fluorescence (blue signals), the samples were excited using a 405 nm laser and detected at 420–520 nm. For yellow fluorescence signals (positive signals), a 514 nm exciter was used, and the signals were monitored from 530–560 nm. The autofluorescence of chloroplast (red signals) was detected in the spectral range of 600–720 nm with an exciter set at 514 nm. The blue, yellow, and red fluorescence were sequentially imaged with a bright field (BF) image obtained simultaneously under the condition of 405 nm excitation.

The protoplast samples were initially examined under the LSCM objective (63×), and then the visual field was zoomed in accordingly. The live image was scanned at an image resolution of 512 × 512, allowing for instantaneous refreshing to changes in the visual field and other parameter settings. However, for capturing an image, a higher resolution of 1024 × 1024 was set with scanning lines averaged three times. The laser intensity was adjusted based on the fluorescence brightness. A photomultiplier tube detector was utilized when the signal intensity was sufficiently high. In cases where necessary, a hybrid detector, frame accumulation of three times, or increased pinhole values were used. All experiments were repeated at least three times. The data were compared and analyzed using SPSS software (version 13.0). Histograms and line graphs were generated using SigmaPlot (version 10.0).

## Results

### Interference caused by chloroplast fluorescence

At present, BiFC systems have been developed in various species including *Arabidopsis*, tobacco, rice, parsley, and others (Table 1). Leaves are commonly used as materials for protoplast preparation, but other organs such as roots have also been utilized. YFP is frequently used as the tag protein, although proteins of other colors such as red, green, and cyan have also been applied. In addition to monochrome fluorescence proteins, multicolor fluorescence proteins have been employed to study protein interactions (Table 1). Fluorescence dyes such as DAPI are commonly used for nuclear localization. Additionally, studies have also utilized tagged fluorescence proteins like GFP, mCherry, and RFP to localize the nucleus, as they have distinct spectra and can be easily distinguished from the target signals. Moreover, BF images are often used to indicate intracellular positioning. The above research provides a technical foundation for this article.

**Table 1:** Case studies of protein interactions using the protoplast BiFC system

Color	Fluorescence protein	Excitation (nm)	Emission (nm)	Microscopy	Protoplast source	Observation of chloroplast autofluorescence	Nucleus localization	References
Red	RFP	549	570	LSCM	Tobacco BY2	No	–	Jach et al. (2006)
	RFP	558	583	LSCM	Tobacco	No	BF/GFP	Li et al. (2012)
Yellow	YFP	514	530–560	LSCM	Rice	Yes	BF	Zhang et al. (2011)
	YFP	480–520	505–565	FL	Parsley	No	CFP	Stolpe et al. (2005)
	YFP	488	505–530	FL	*Arabidopsis*	Yes	BF	Wu et al. (2009)
	YFP	Single laser	–	LSCM	Tobacco	Yes	BF	Citovsky et al. (2006)
	EYFP	480	500–550	LSCM	*Arabidopsis* root	Yes	mCherry/BF	Dervisi et al. (2022)
	EYFP	488	505–565	LSCM	*Arabidopsis*	No	BF	Olejnik et al. (2011)
	YFP	514	–	LSCM	*Arabidopsis*	Yes	BF	Zhang et al. (2016)
	YFP	480	500–550	LSCM	*Arabidopsis*	No	BF	Hussain et al. (2018)
	YFP	–	–	LSCM	*Arabidopsis*	Yes	DAPI	Chang et al. (2019)
	nVenus + cCFP	–	–	LSCM	*Arabidopsis*	Yes	DAPI/BF	Liu et al. (2021)
	nVenus + cCFP	488	500–530	FL/LSCM	Tobacco BY2	No	mCherry/BF	Lee et al. (2008)
Green	GFP	450–490	520–560	FL	Rice	Yes	BF	Chen et al. (2006)
	GFP	–	450–490	FL/LSCM	*Freesia/Arabidopsis*	No	RFP	Shan et al. (2019)
	GFP	488	500–530	LSCM	Rice	Yes	BF	Zhang et al. (2011)
	GFP	–	–	LSCM	*Arabidopsis*	Yes	DAPI/BF	Liu et al. (2021)
Cyan	nCerulean + cCFP	458	480–520	FL/LSCM	Tobacco BY2	No	mCherry/BF	Lee et al. (2008)
	nCerulean + cCFP	–	–	LSCM	*Arabidopsis* root	Yes	BF	Dervisi et al. (2022)

*LSCM* laser scanning confocal microscope, *FL* fluorescence microscope, *BF* bright field

**Fig. 1:**
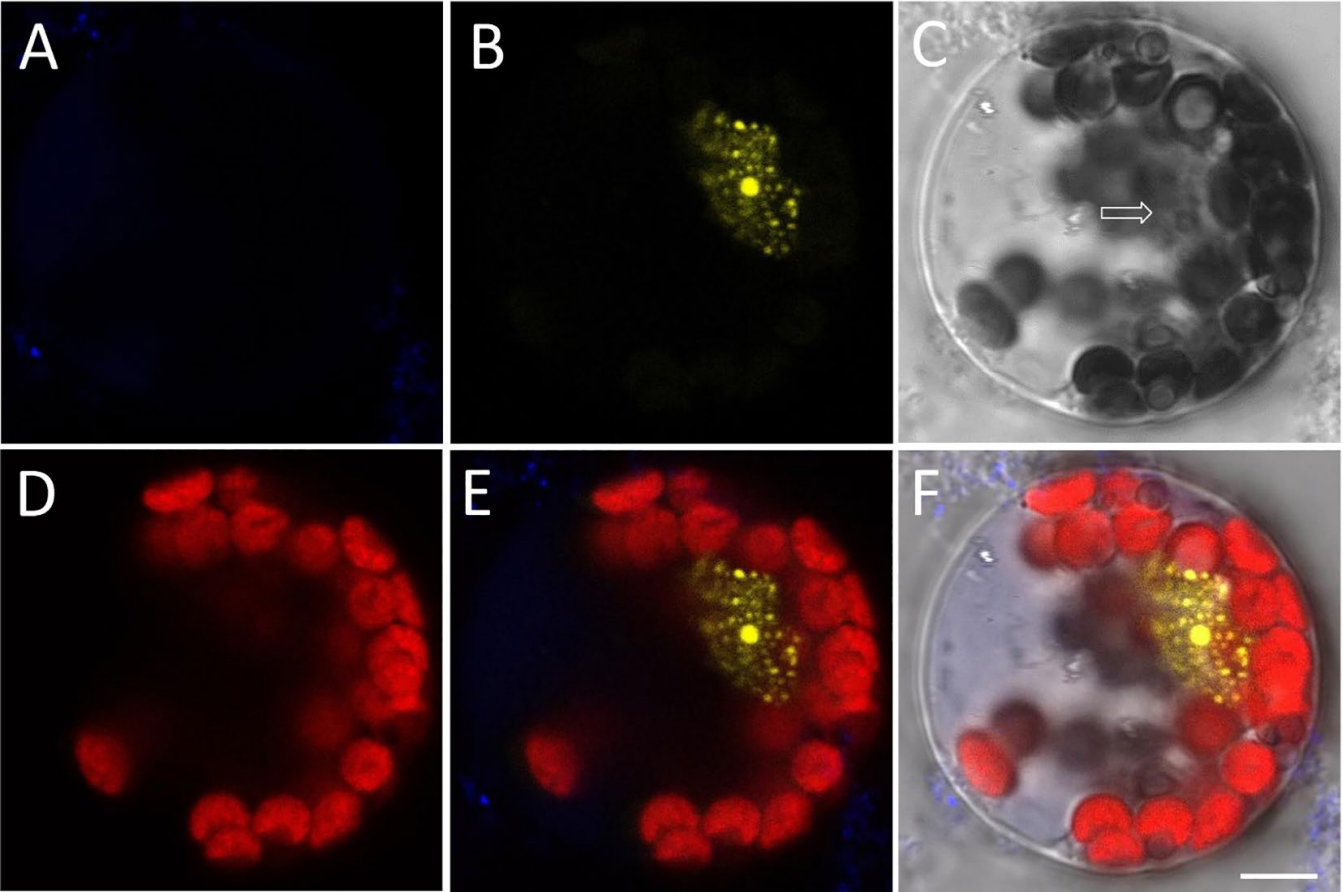
BiFC signal imaging in protoplast with hardly observed DAPI signal. Gene constructs were co-transfected into *Arabidopsis* protoplasts to detect their intracellular interaction and localization. **A** DAPI fluorescence (blue), used as a nuclear marker. **B** YFP fluorescence (yellow) produced by the BiFC assay. **C** Bright filed image of the protoplast. Arrow shows the nuclear area. **D** Autofluorescence (red) of chloroplasts. **E** Merged image of **A**, **B** and **D**. **F** Merged image of **A**–**D**. All images are shown at the same magnification. Scale bar = 5 µm

**Fig. 2:**
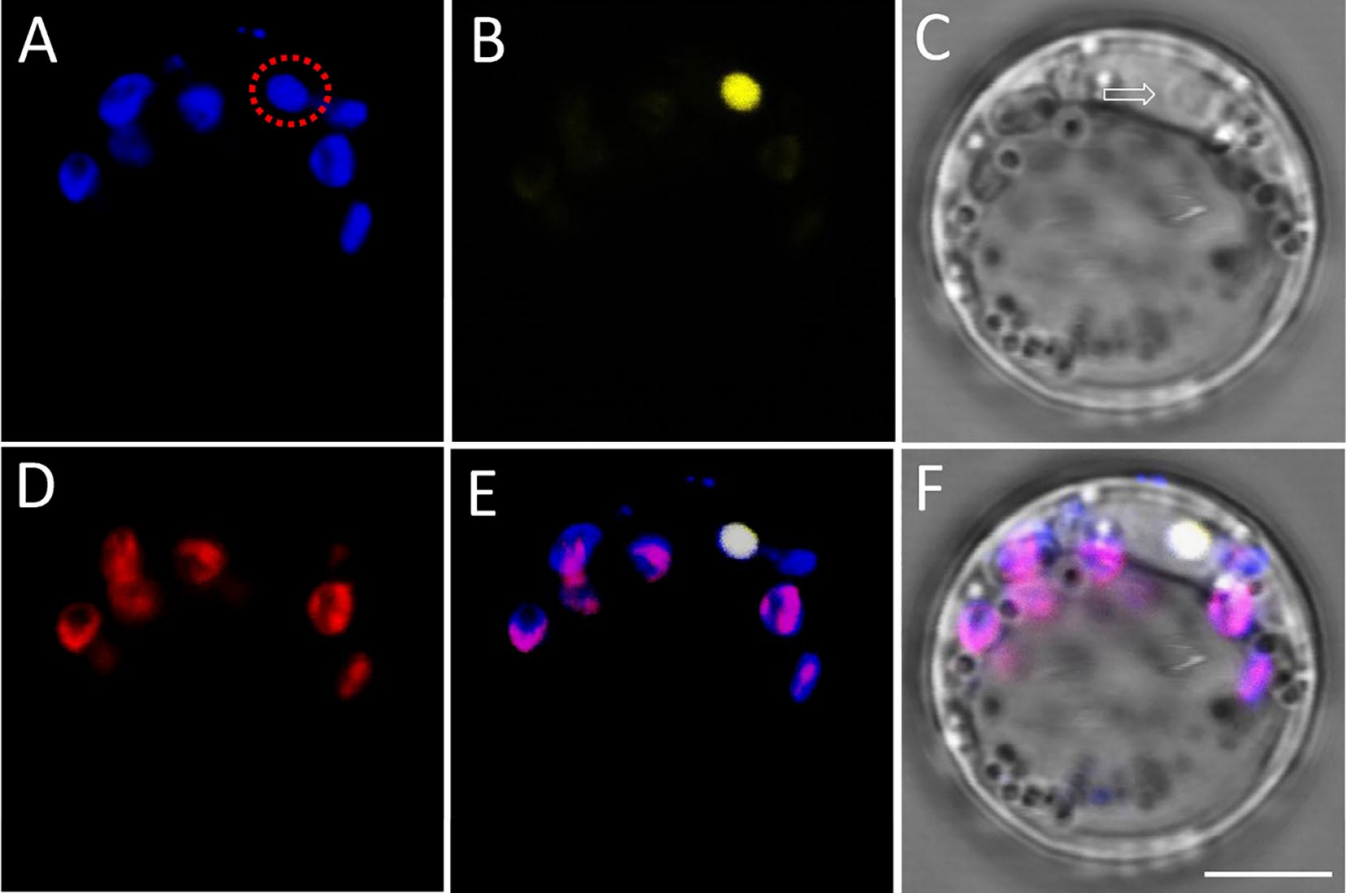
BiFC signal imaging in protoplast with weak DAPI signal that appeared concurrently with chloroplast signal. **A** Fluorescence of DAPI and chloroplasts. **B** YFP fluorescence. **C** Bright filed image of the protoplast. Arrow shows the nuclear area. **D** Autofluorescence of chloroplasts. **E** Merged image of **A**, **B** and **D**. **F** Merged image of **A**–**D**. Dotted circle shows the DAPI signals from the nuclear area, which is inferred from comparison of signals from (**A**) and (**D**). All images are shown at the same magnification. Scale bar = 5 µm

**Fig. 3:**
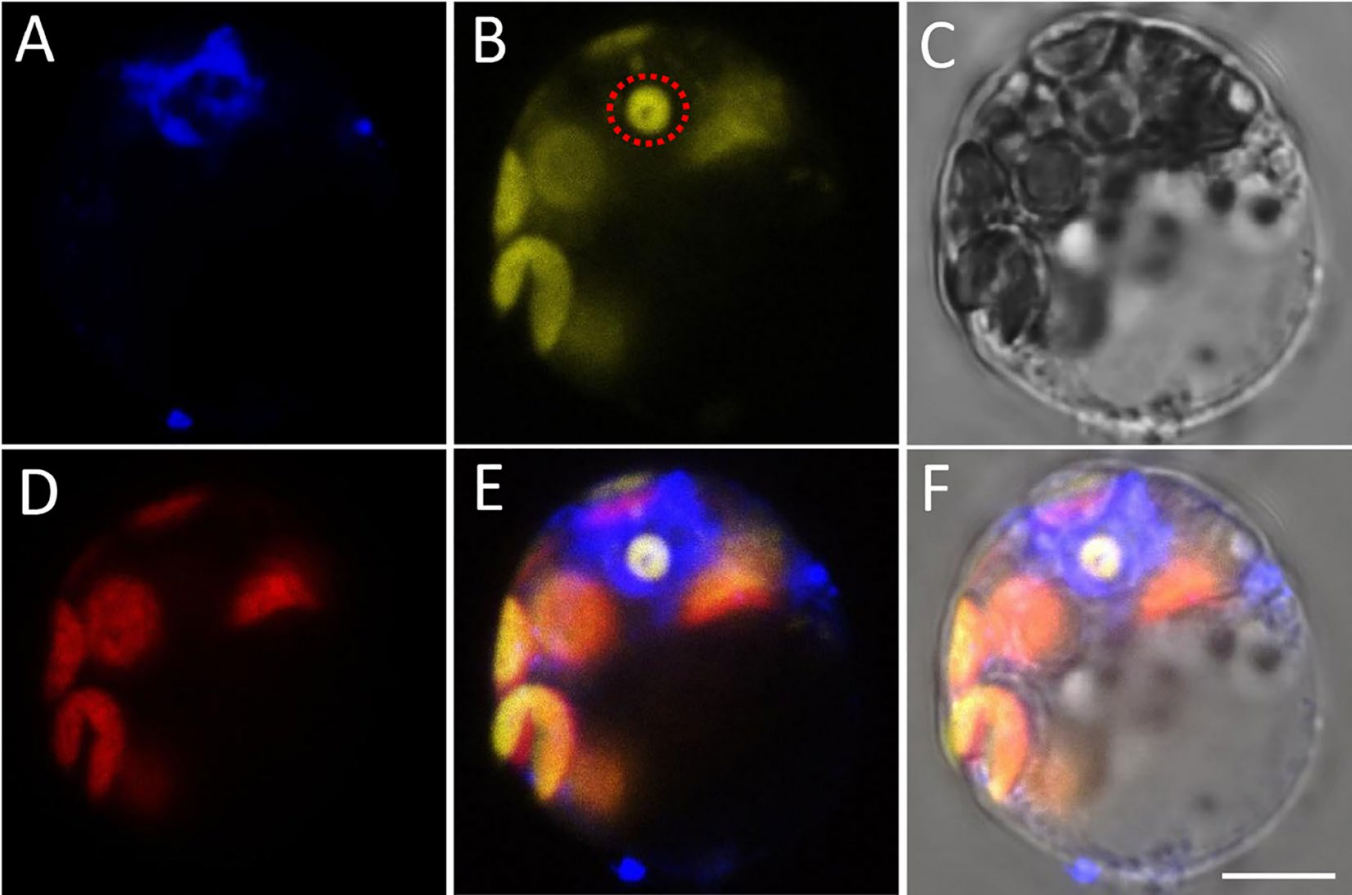
Protoplast with weak BiFC signal that appeared concomitantly with chloroplast signal. **A** DAPI fluorescence. **B** Fluorescence of YFP and chloroplasts. **C** Bright filed image of the protoplast. **D** Autofluorescence of chloroplasts. **E** Merged image of **A**, **B** and **D**. **F** Merged image of **A**–**D**. Dotted circle shows signals from the nucleolus. All images are shown at the same magnification. Scale bar = 5 µm

**Fig. 4:**
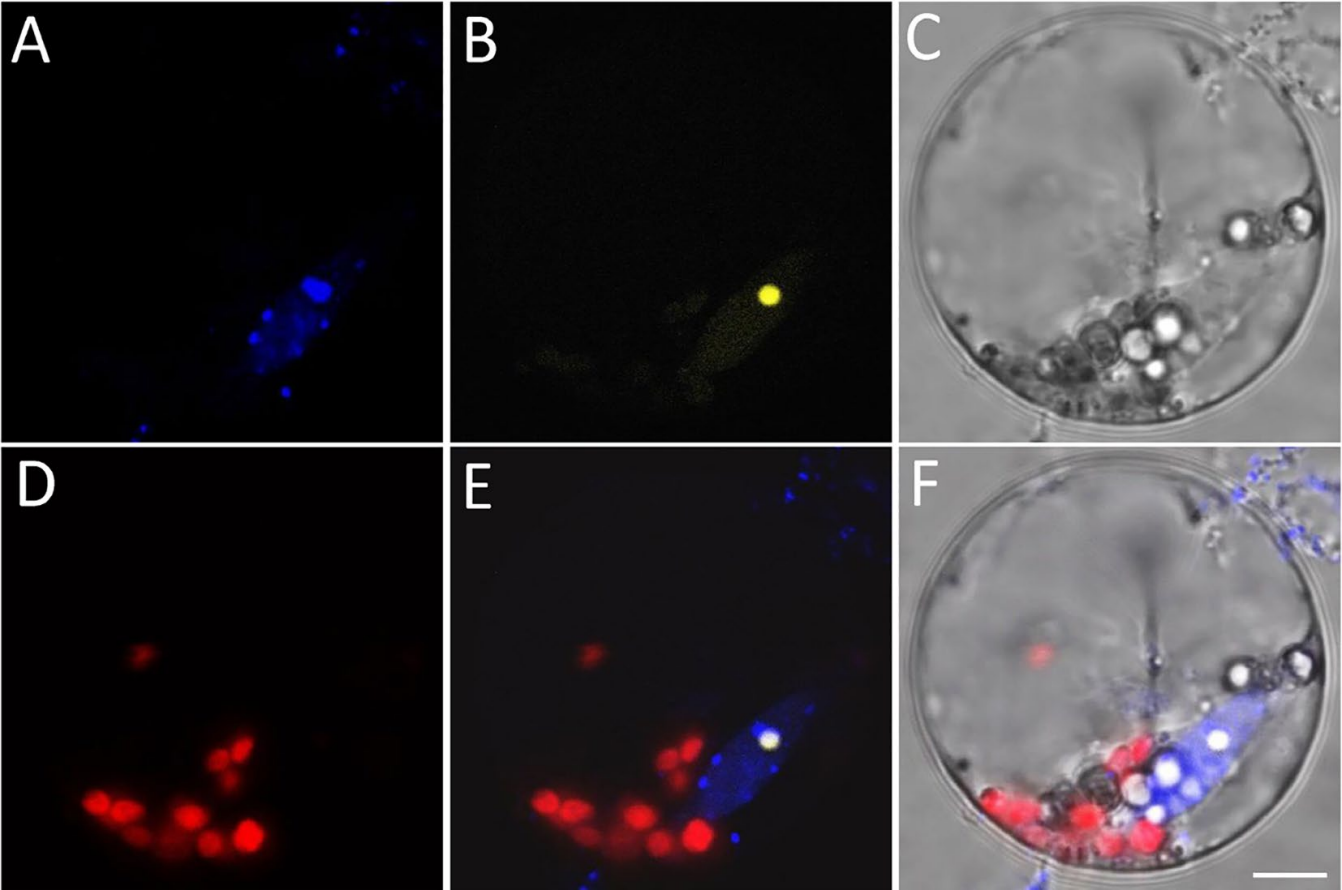
Protoplast with strong signals of both BiFC and DAPI. **A** DAPI fluorescence. **B** YFP fluorescence. **C** Bright filed image of the protoplast. **D** Autofluorescence of chloroplasts. **E** Merged image of **A**, **B** and **D**. **F** Merged image of **A**–**D**. All images are shown at the same magnification. Scale bar = 5 µm

The *Arabidopsis* protoplasts often contain numerous chloroplasts, which can cause background noise due to their strong autofluorescence after excitation. In this study, we examined the fluorescence signals at different intensities (Figs. 1, 2, 3, 4). The images for BiFC localization can be categorized into four types: in the first type, the DAPI fluorescence was barely observed (Fig. 1); in the second type, the DAPI signals were weak and appeared concomitantly with the chloroplast signal (Fig. 2); in the third type, the positive signals were weak and appeared alongside the chloroplast signal (Fig. 3); and in the fourth type, both the DAPI signals and the positive signals were strong (Fig. 4).

In the first type of situation, the DAPI signal is quite weak, and the nuclear region is only barely distinguishable from chloroplasts (Fig. 1B–D). Since the DAPI signals are not visible in the corresponding nuclear area (Fig. 1A), we can only speculate that the two genes may interact at the nucleolus area (Fig. 1B–F). A high concentration of DAPI has the ability to stain live cells similarly to dead ones (Digby et al. 2019). In this situation, the DAPI signal intensities could be enhanced by increasing the staining time or using a higher concentration of dye.

Figure 2 illustrates a situation where DAPI signals are still weak and the localization of the nucleus may be disturbed by chloroplast signals. When exciting DAPI fluorescence, the blue signals generally overlap with the signals of the chloroplasts (Fig. 2A and D). However, there is one particle that overlaps with the area of the YFP signal (Fig. 2A, B). Under BF microscopy, the outlines of the nuclear region and the chloroplast are visible (Fig. 2C). In this scenario, the intensity of DAPI signals can be increased for better imaging by adjusting the laser illumination efficiency or other exposure parameters (Fig. 2A, enclosed area). However, it should be noted that the autofluorescence of the chloroplast also increases after these adjustments, as shown by the signals outside the enclosed area in Fig. 2A. If the DAPI fluorescence region is similar in size to the chloroplast, the DAPI signals may be easily mistaken for autofluorescence signals from the chloroplast (Fig. 2D and E). This could potentially lead to false negative conclusions without the reference of chloroplast autofluorescence. However, we can still figure out that the two genes interact at the nucleolus region by using autofluorescence of chloroplasts as a reference (Fig. 2D).

Sometimes, the yellow epifluorescence may appear faint when viewed through the eyepieces due to weak fluorescence, specific focusing Z position, or fluorescence quenching. However, the intensity of the yellow signals can be increased for photography by adjusting the software parameters, resulting in enhanced chloroplast signals at the same time. Figure 3 illustrates this situation when positive signals and chloroplast fluorescence can be observed simultaneously under the photographic conditions for YFP signals (Fig. 3B). The image of the chloroplast autofluorescence (Fig. 3D), along with images of BF (Fig. 3C) and DAPI signals (Fig. 3A), could lead to a misconception that interaction occurs in both chloroplasts and nuclei. This situation reminds us that there is overlap between chlorophyll emission spectrum and YFP, and a comprehensive interpretation of the results must be conducted by combining the predicted subcellular localization information of the interested protein and multiple BiFC samples.

Figure 4 illustrates the imaging scenario where both DAPI and positive fluorescence signals are strong. The two target genes appear to interact with each other and potentially localize at the nucleolus. From this case, it can be seen that to obtain a satisfactory image for localization, it is crucial that the protoplast's shape remains intact, each type of fluorescence exhibits strong intensity, and both the fluorescent and BF channels are accurately focused at the same Z position.

### Analyzing the autofluorescence spectrum of *Arabidopsis* chloroplasts

In previous studies, there was a wide variation in the excitation and emission wavelengths used to detect chloroplast autofluorescence. It has been reported that *Arabidopsis* plant tissues emit the highest autofluorescence at approximately 590–600 nm (Ohad et al. 2007). To investigate the autofluorescence of *Arabidopsis* protoplasts, previous studies have frequently employed blue or green light as the excitation source. For instance, fluorescence was excited using wavelengths of 488 nm or 514 nm, with the emission spectrum being monitored in the range of 650–750 nm (Zhang et al. 2011); blue lasers with a wavelength of around 470 nm were utilized for excitation, while the emitted fluorescence in the range of 680–700 nm was detected (Dervisi et al. 2022); or 488 nm wavelength was used for excitation, and the detection spectrum was set to 650–710 nm (Wu et al. 2009).

**Fig. 5:**
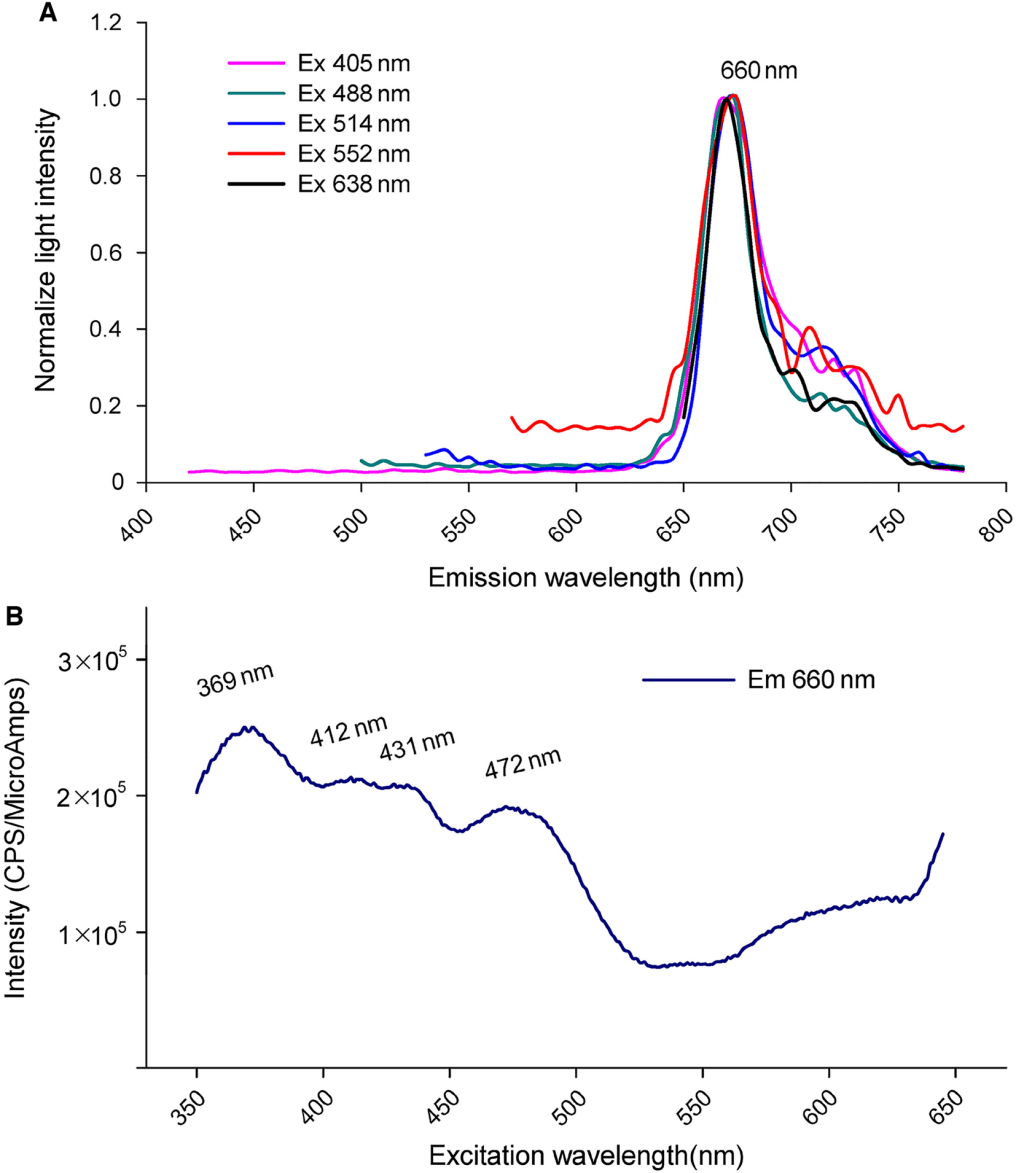
Emitting spectra of autofluorescence from *Arabidopsis* protoplast chloroplasts and excitation spectra of autofluorescence from protoplast suspension. **A** Chloroplast particles were excited using five laser exciters: 405 nm, 488 nm, 514 nm, 552 nm and 638 nm. The fluorescence signals were detected starting from 420 nm, 500 nm, 530 nm, 570 nm and 650 nm, respectively, and ending at 790 nm. The spectrum width was 25 nm and the step width was 5 nm. Each test was repeated at least six times. **B** The autofluorescence spectra of *Arabidopsis* protoplast suspension were monitored using a fluorescence spectrometer with the emission wavelength set at 660 nm

To avoid detection of extraneous signals from chloroplast autofluorescence that cause spectral overlapping with interested signals and background noise, it is important to have a clear understanding of its spectra in order to appropriately set the detection spectrum. We examined the emission spectrum of chloroplast autofluorescence using the spectra scanning module of the software. The chloroplast particles were excited by five different lasers (405 nm, 488 nm, 514 nm, 552 nm, and 638 nm), and the fluorescence intensity was measured across a wide spectral band. The results demonstrated that all the lasers were capable of exciting fluorescence from chloroplasts. The strongest chloroplast autofluorescence was observed between 625 and 750 nm, with peak fluorescence signals present at 660 nm (Fig. 5A).

To further investigate the autofluorescence of protoplasts, we conducted a fluorescence spectrum analysis of the protoplast suspension using a fluorescence spectrometer (Fig. 5B). When set emission wavelength at 660 nm, a relatively higher intensity of autofluorescence with an excitation wavelength ranging from 350 nm to around 475 nm could be stimulated from the protoplast suspension (Fig. 5B).

In summary, our results demonstrated that chloroplasts can be stimulated and emit fluorescence across a broad range of wavelengths, thus causing the spectra of DAPI (blue) and BiFC signals (yellow) to partially overlap with the wavelength range of chloroplasts. When their signals were weak, it became challenging to distinguish them from the background signals generated by chloroplast autofluorescence.

### Impact of pipetting on cellular intactness of protoplast

**Fig. 6:**
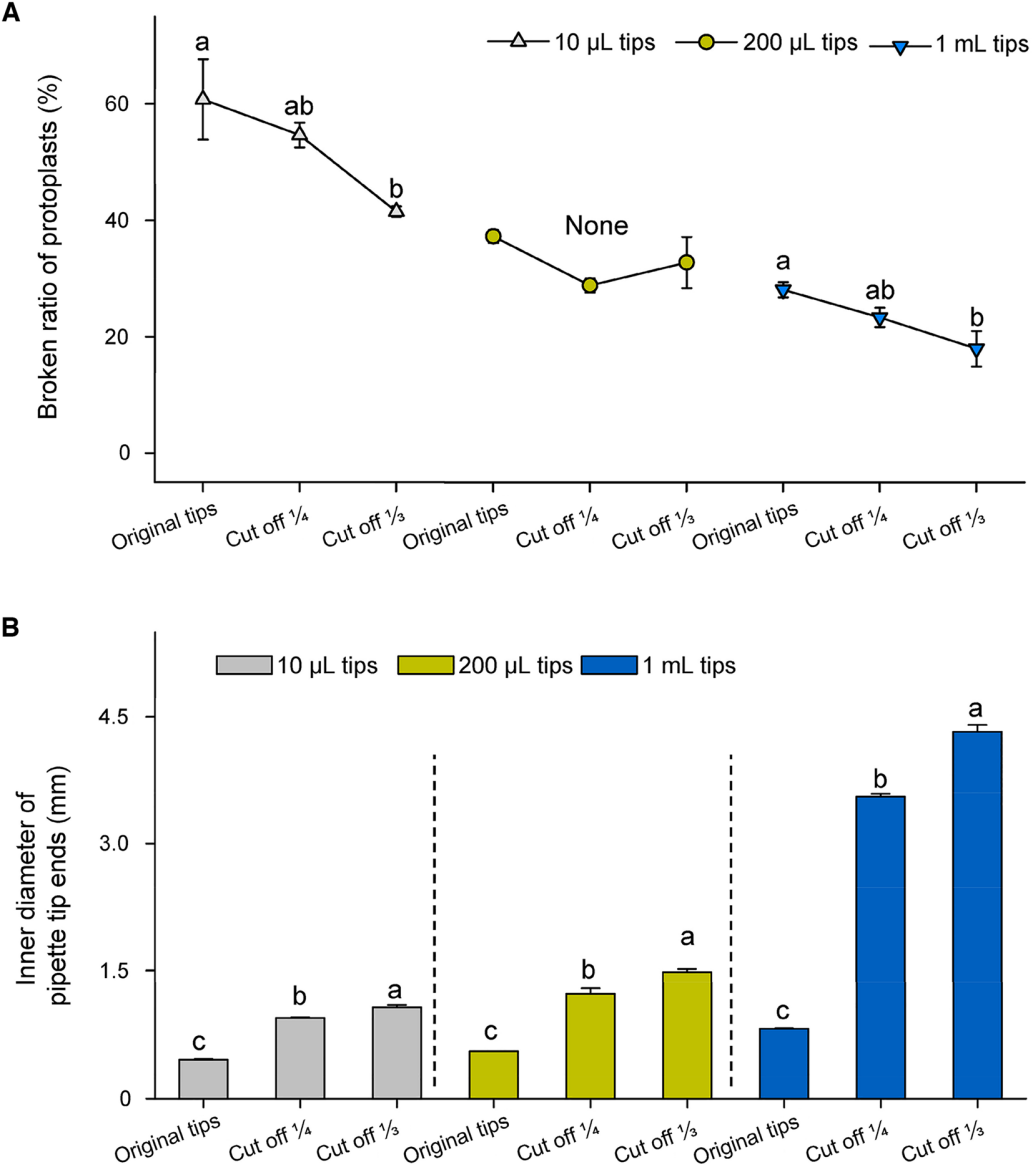
The correlation between inner diameter of pipette tips and protoplast intactness. **A** The broken ratio of protoplasts decreased when protoplast samples were pipetted using tips with cut ends. **B** The inner diameter of different tip ends. The broken ratio of protoplasts was calculated using the equation: broken protoplasts/total protoplasts. Protoplast samples were pipetted at a speed of 2–3 s per pipetting action. The test was repeated three times

**Fig. 7:**
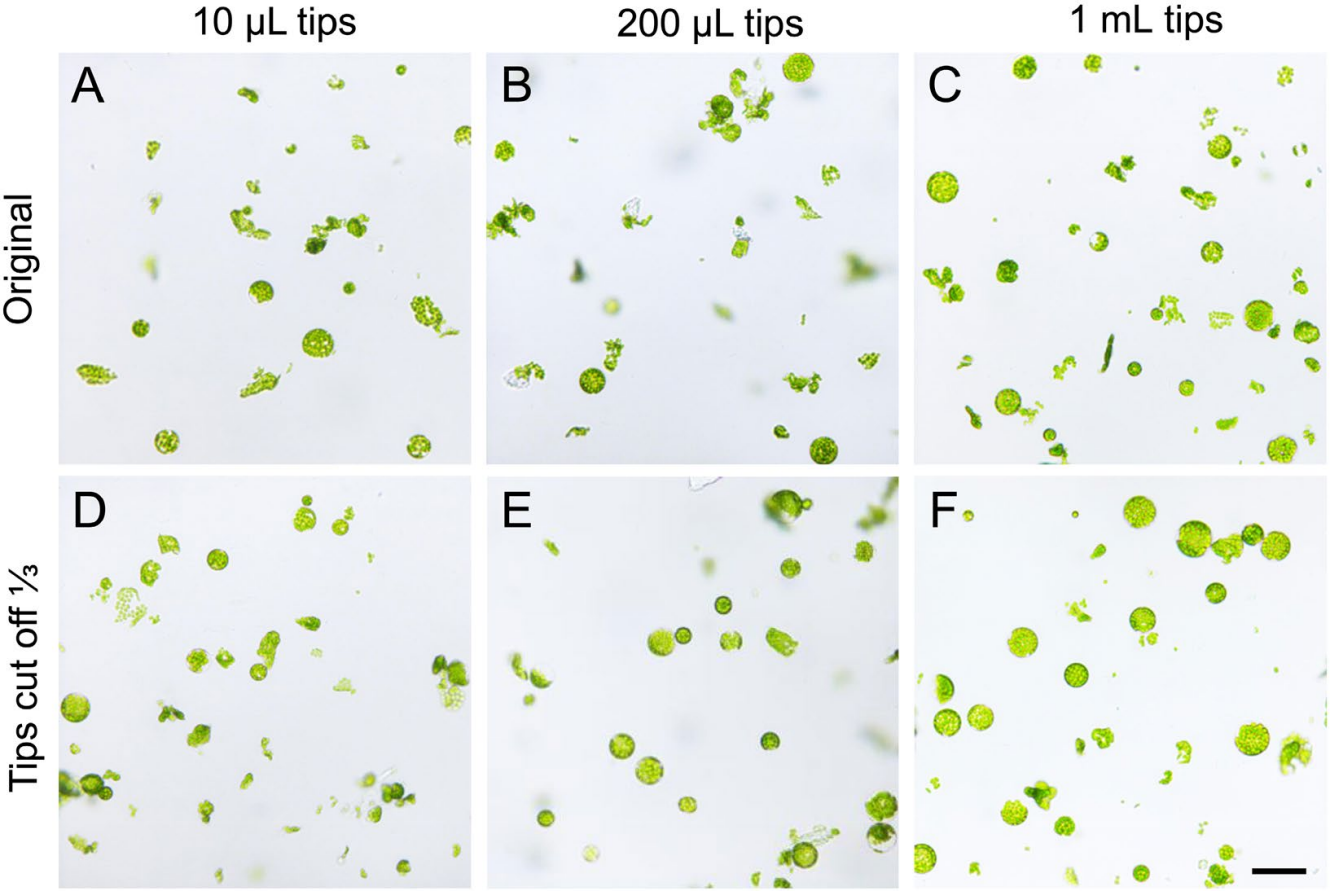
Light microscopic imaging of protoplasts pipetted with different tips. Protoplasts (25 µL, or a droplet of sample solution) were examined under an inverted microscope with a 20× objective. Images **A**–**C** were samples pipetted with original tips of 10 µL, 200 µL and 1 mL, respectively, while images **D**–**F** were the samples pipetted with the corresponding tips with ends cut one-third. All images are shown at the same magnification. Scale bar = 50 µm

The common types of pipette tips had inner diameters much larger than the protoplasts, with an average diameter of around 24.6 μm. Even the 10 μL tips had a diameter of around 0.45 mm. Theoretically, protoplast samples can be easily handled with any commonly used pipette tips (Yoo et al. 2007). However, with the increase of the inner diameter of pipette tip ends (Fig. 6B), the broken ratio of protoplasts decreased (Fig. 6A). Our results indicated that pipette tips with partially cut ends were able to preserve the protoplast shape more effectively compared to the original tips (refer to Figs. 6, 7). A previous study also suggested that using an auto-pipette with a low speed or tips with a larger orifice would yield better results (Planchais et al. 2023). This might be because a larger sucking and pushing speed of the pipette could bring stronger mechanical stress on the cells. If the speed of the pipette remains constant, the smaller the cross section of the pipette tips, the greater the pressure on the protoplasts. As a result, they become more vulnerable to breakage, leading to a decrease in the intactness ratio.

### Protoplast sample preparation for LSCM and observation

**Fig. 8:**
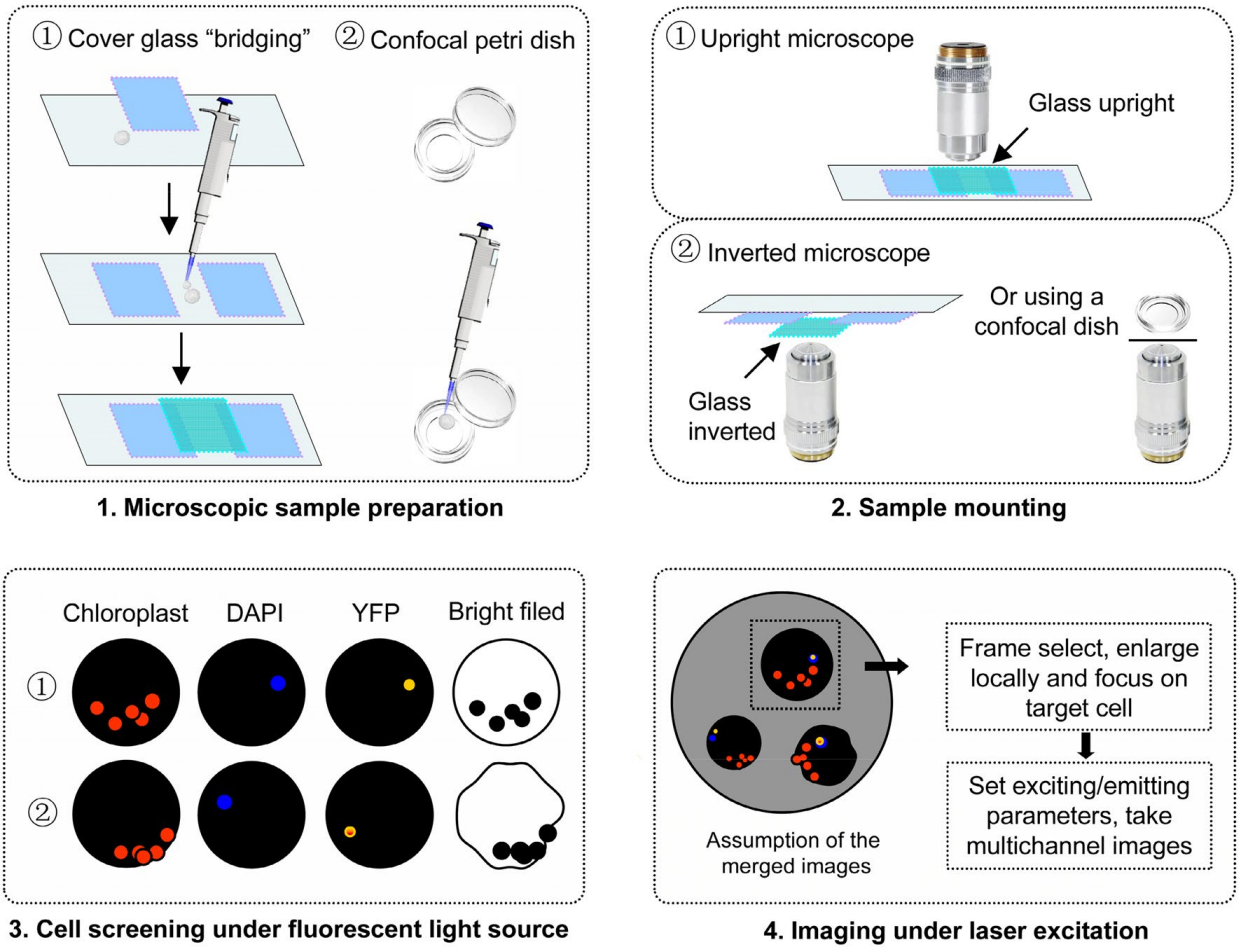
Illustration of preparation and observation of protoplast using LSCM. Directly mounting the samples between the cover slide and the glass slide is not preferred, as it may cause the cells to be compressed due to the weight of the cover glass or excessive downward movement of the objective lens. To avoid this, a “bridge” made by cover glasses or a confocal petri dish are employed to protect samples (1). The “bridging” method is suitable for inspection under an upright microscope or an inverted microscope, or a confocal dish can be used under an inverted microscope (2). Protoplast samples were mounted, examined and screened based on their appearance under multiple fluorescence channels and bright field conditions (3). Next, selected protoplasts are imaged using lasers as the excitation source (4)

The workflow for observing protoplasts using LSCM is illustrated in Fig. 8. Initially, the protoplast sample is prepared on a temporary slide. It is important to note that it is not preferred to directly mount the samples between the cover slide and the glass slide, as this may cause the cells to be compressed due to the weight of the cover slide or excessive downward movement of the objective lens. To avoid this, the samples are prepared using a “bridging” method as described below: 10 μL of water (< 15 μL) is added to each end of the glass slide, cover glasses was placed on top of the water droplets, 45 μL of the sample (< 55 μL) is added in the gap (width ~ 1.1 cm) between the two cover glasses, and then a third cover glass was placed between and on top of the two cover glasses (Fig. 8-1). The temporary bridging slide is suitable for inspection under an upright microscope with an objective magnification of up to 40× due to space limitations on the microscope stage. However, this slide can be used with a higher magnification objective, such as 60×, when an inverted microscope is utilized. In such cases, the slide should be placed upside down, with the cover glass facing the lens hole (Fig. 8-2). If a universal specimen holder, commonly found in inverted microscopes, is used, it is recommended to use a confocal petri dish to prepare the protoplast sample. This dish has a cover-glass-like bottom and prevents the protoplasts from being compressed.

Next, in order to identify cells suitable for imaging, protoplasts are examined based on their appearance under multiple fluorescence channels and transmitted-light illumination conditions. Initially, a halogen lamp is used to focus and inspect the sample under the oculars of the LSCM microscope. Subsequently, lasers are employed for capturing images. When exposed to the halogen lamp, the fluorescence emitted by the nucleus, fluorescent proteins, and chloroplasts exhibits different colors. By observing the intensity and localization of each specific fluorescence color under multiple channels, potential candidate protoplasts can be identified. For instance, if a protoplast emits a fluorescence that is of interest, it can be inferred that there is a possibility of a specific interaction occurring in the nucleus area if positive signals appear to overlap with nucleus markers, and can be easily distinguished from chloroplast signals. Additionally, under transmitted-light, the candidate cell needs to be round and intact without any leakage of internal contents (e.g. chloroplasts).

Finally, the observation is shifted from using eyepieces (with a halogen lamp as the light source) to utilizing software, using lasers as the excitation source. This allows for the selection and local zooming in the desired protoplast, resulting in only one cell being visible in the viewing window. Once the Z position has been finely adjusted and the imaging parameters have been set, the targeted protoplast is prepared to be captured as multiple images with distinct fluorescence.

## Discussion

### Subcellular localization prediction based on the results of fluorescence and BF images

We observed that protoplasts with intact shape tended to emit weaker DAPI signals compared to cells with compromised integrity. As a result, imaging DAPI signals in live protoplasts became more challenging. In such cases, the study could use a sensible detector or adjust other parameters to ensure optimal signal intensity detection. It is worth noting that chloroplast autofluorescence has a broad emission spectrum. As a result of this manipulation, DAPI and chloroplast signals may appear simultaneously, as chloroplasts can emit a slight fluorescence in the 420–520 nm range. Similarly, when YFP fluorescence is faint, such adjustments may also lead to the concurrent presence of chloroplasts and YFP signals.

To prevent false positive conclusions resulting from the self-assembly of fluorescence protein halves, it is advisable to employ a quantification that can be compared against the signal-to-noise obtained from a suitable negative control (Horstman et al. 2014). However, the intensity of fluorescence in certain protoplasts may be weak, posing challenges in accurately assessing and quantifying positive cells. Chloroplasts can also serve as reliable internal markers for effectively distinguishing other signals. Utilizing autofluorescence and BF images of chloroplasts as references can be beneficial in reducing interference in subcellular localization.

It is recommended to capture BF protoplast images at a Z position where both the round shape of the protoplast and the outline of the nucleus are in focus. It is important to note that the focal plane for BF imaging may not align with the position of strongest multichannel fluorescence. Cells have a certain Z depth, and the optimal Z position for capturing fluorescence images (reflected signal) may differ from that for capturing appropriate BF images (transmitted signal). Additionally, when observing chloroplasts under BF mode, they may produce a dark background that can interfere with the detection of the nucleus area. In such cases, capturing a series of confocal light sections and generating a projected fluorescence image can be beneficial (Olejnik et al. 2011; Shumskaya et al. 2020; Wu et al. 2009).

### Settings of the excitation and emission spectrum

Sample inspection is greatly impacted by the spectrum setting. The fluorescence signal can be monitored more effectively when the parameter setting matches the spectrum of the fluorescence protein. In epifluorescence microscopes, the excitation and emission settings are directly used by selecting filters that correspond to a specific fluorescence channel. However, in the case of LSCMs, both the excitation and detection wavelength ranges can be adjusted.

To enhance imaging efficiency, the detection range of fluorescence in LSCM can be optimized based on the characteristics of the fluorescent substances. The autofluorescence of chloroplast is strong, stable, and has a wide emitting spectrum. Consequently, the imaging settings for chloroplast varied across different studies (Dervisi et al. 2022; Ohad et al. 2007; Zhang et al. 2011). Therefore, if the fluorescence of interest is weak, the detection spectrum can be expanded to enhance the fluorescence intensity. Conversely, if the signals of interest are strong or if there is cross-color interference due to chloroplast autofluorescence, the detection spectrum can be narrowed to mitigate these effects.

### Retaining sample intactness when prepare samples for inspection

During the preparation process of protoplasts, gentle manipulation is beneficial for maintaining their integrity (Planchais et al. 2023; Yoo et al. 2007). In regards to the final step of sample treatment, although the inner diameters of the pipette tips were larger than the size of the protoplasts, cell integrity of the protoplast sample was better preserved when they were gently pipetted using cut tips with larger apertures. Additionally, the tips' ends can be treated to smooth the cut edge by briefly exposing them to the external flame of an alcohol lamp for less than 1 s. We found that this treatment will not significantly affect their inner diameters.

In addition, it is important to avoid squeezing the protoplasts. The direct preparation method using a glass slide and cover slide can lead to squishing of the protoplasts, which significantly impacts the integrity of the samples. A recommended approach is to use the “bridging” method, which creates a space of 0.17 mm in height (equivalent to the thickness of the cover slide) to accommodate the protoplasts and prevent squeezing. Alternatively, the samples can be observed using confocal dishes under an inverted microscope.

### Other factors affecting imaging

The cell status of protoplasts, being spherical, active, and vulnerable, can undergo significant changes that can greatly impact the photographic outcome. The movement of glass slides or the objective lens might cause the cover glass, which is attached to the immersion oil on the lens, to also move, resulting in a shift in the position of the protoplasts. Therefore, it is important to handle both the sample slide and objective lens gently to prevent drastic changes in the protoplast position. Additionally, it is advisable to minimize repeated or prolonged exposure of samples to exciter light as protoplasts are prone to losing their integrity, changing position, and diminishing fluorescence.

To improve experiment efficiency, cells displaying interesting phenotypes can be visually screened under the eyepieces before capturing images using software. Candidate protoplasts can be identified based on factors such as cell integrity, cell shape, and localization of each fluorescence color. However, advancements in microscopy have significantly reduced the background noise produced by autofluorescence (Digman et al. 2008). Nowadays, with the use of advanced instrument models, samples can be automatically screened and imaged in the software without the need for manual inspection under the eyepieces (Cao et al. 2023). Developed technologies such as fluorescence lifetime imaging microscopy and methods for separating overlapping spectral lines can effectively eliminate the noise caused by autofluorescence (Digman et al. 2008; Kodama 2016; Zhao et al. 2020).

## Data Availability

Agree.
